# Characteristics, Roles and Applications of Proteinaceous Elicitors from Pathogens in Plant Immunity

**DOI:** 10.3390/life13020268

**Published:** 2023-01-18

**Authors:** Zhangqun Li, Junnan Liu, Wenting Ma, Xiaofang Li

**Affiliations:** 1 School of Pharmaceutical Sciences, Taizhou University, Taizhou 318000, China; 2 Institute of Biopharmaceuticals, Taizhou University, Taizhou 318000, China; 3 School of Life Science, Taizhou University, Taizhou 318000, China

**Keywords:** elicitors, immune response, harpin, NLPs, elicitin

## Abstract

In interactions between pathogens and plants, pathogens secrete many molecules that facilitate plant infection, and some of these compounds are recognized by plant pattern recognition receptors (PRRs), which induce immune responses. Molecules in both pathogens and plants that trigger immune responses in plants are termed elicitors. On the basis of their chemical content, elicitors can be classified into carbohydrates, lipopeptides, proteinaceous compounds and other types. Although many studies have focused on the involvement of elicitors in plants, especially on pathophysiological changes induced by elicitors in plants and the mechanisms mediating these changes, there is a lack of up-to-date reviews on the characteristics and functions of proteinaceous elicitors. In this mini-review, we provide an overview of the up-to-date knowledge on several important families of pathogenic proteinaceous elicitors (i.e., harpins, necrosis- and ethylene-inducing peptide 1 (nep1)-like proteins (NLPs) and elicitins), focusing mainly on their structures, characteristics and effects on plants, specifically on their roles in plant immune responses. A solid understanding of elicitors may be helpful to decrease the use of agrochemicals in agriculture and gardening, generate more resistant germplasms and increase crop yields.

## 1. Introduction

Plants, the main nutrient sources for humans, are attacked by many pathogens that can consume them, devastating yields and causing economic losses, which make pathogen infection among the most formidable challenges to global food security. To reduce crop loss, many chemical bactericides and fungicides have been used to prevent or treat diseases caused by pathogens. However, in addition to inducing toxicity in pathogens, these conventional agrochemicals pose hazards to the environment and human health [1]. Furthermore, continuous applications of conventional germicides may fail because of the emergence of resistant pathogens. Recent advances in the study of plant innate immunity, especially plant-pathogen interactions, have shown that elicitors can trigger immune responses in plants that lead to intense and persistent resistance to pathogens [2,3,4,5,6]. The application of elicitors seems to be an attractive and promising method for treating agricultural pathogens in a green and environmentally friendly manner.

Advances in research into plant innate immunity have revealed that plants employ a multilayered surveillance system to detect and respond to pathogen invasion. According to the typical zigzag model, there are two layers in plant innate immunity: namely, pattern-triggered immunity (PTI) and effector-triggered immunity (ETI) [7]. In PTI, plant pattern recognition receptors (PRRs) on the plasma membrane recognize pathogen-/microbe-associated molecular patterns (PAMPs/MAMPs) and trigger subsequent immune responses, such as membrane depolarization, calcium influx, a spike in reactive oxygen species (ROS) levels, activation of mitogen-activated protein kinase (MAPK) cascades, induction of pathogenesis-related (PR) gene expression and callose deposition [3]. PAMPs/MAMPs are highly conserved molecular signatures, including peptides such as flagellin (flg22) and elongation factor Tu (EF-Tu, elf18) from bacteria, elicitins from oomycetes, coat proteins and RNA replicases from virus, carbohydrates such as chitin from fungi and peptidoglycan (PGN) from bacteria, and ceramides such as Pi-Cer D from oomycetes. To suppress plant PTI and facilitate invasion, many pathogens have evolved PTI-suppressive mechanisms termed effector-triggered susceptibility (ETS). To prevent and survive pathogen invasion, plants recognize effectors by nucleotide binding (NB) and leucine-rich repeat (LRR) proteins and induce robust ETI, causing hypersensitive cell death (HR). New effectors and NB-LRR proteins evolved to induce new ETI again [7]. Associations between PTI and ETI have been reported by several excellent studies. PTI and ETI share several metabolites, e.g., glutathione and neodiosmin and signaling pathways, e.g., ROS spike, Ca^2+^ burst, activation of MAPK cascades and phytohormone pathways [8,9,10].

Elicitors can be classified into endogenous and exogenous categories according to their origins (Table 1). Endogenous elicitors, such as oligo-galacturonic acid and xylan fragments from the plant cell wall, are generated by host plants. Exogenous elicitors, such as chitin, β-glucan, lipopolysaccharide (LPS), harpins, elicitins, virus coat proteins and RNA replicases are produced by pathogens. According to their chemical properties, elicitors can be classified into proteinaceous, carbohydrate, chemical-inducing elicitors, and so on. Exogenous proteinaceous elicitors are proteinaceous elicitors from microorganisms, especially pathogens, and they are involved in many processes, such as the virulence of pathogens, abiotic stresses and biotic stresses in plants [11,12,13,14,15]. Although many studies and reviews have focused their attention on the involvement of proteinaceous elicitors in plant innate immunity and abiotic stresses in past decades, there is a lack of up-to-date reviews on the characteristics and functions of proteinaceous elicitors. In this mini review, we mainly focus on the sources and properties of several important kinds of exogenous proteinaceous elicitors (i.e., harpin, NLPs and elicitins), their involvement and signaling mechanisms in the immune responses in plants and discuss how to apply this knowledge to improve abiotic and biotic stress tolerance in crop plants.

## 2. Harpins

### 2.1. Architectures and Functions of Harpins

Harpins, the first identified cell-free elicitor causing a hypersensitive response (HR) from Gram-negative plant-pathogenic bacteria, are an important type of elicitor that is involved in the interaction between plants and pathogens. They function in many physiological responses in microbes and abiotic and biotic stresses in plants, e.g., working as virulence factors in the invasion of bacterial pathogens into a host, inducing HR and non-HR immunity in plants and promoting plant growth [16,17,18,19]. Harpins are elicitors with approximately 10–60 kDa, encoded by sequences located in the hypersensitive response and pathogenicity (*hrp*) gene cluster and released through the type III secretion system [16,20]. Most harpins are acidic, hydrophilic, and thermostable and are sensitive to chemically denaturing proteins because of their distinctive sequences and architectures [16,19,20,21,22]. Harpins are enriched with glycine and serine but carry few aromatic amino acids and low levels of cysteine, which is an amino acid that contributes to disulfide bonds. The level of glycine exceeds 15% in most harpins, and these glycine residues cluster in specific protein regions [16]. Harpins usually carry many (from two to nine) α-helixes, and most do not harbor domains similar to other proteins in bacteria or plants, which makes them good elicitors in plants [16,23,24,25].

Since the first harpin, HrpN (sometimes called HrpNea) was identified in the fire blight pathogen *Erwinia amylovora*, a devasting pathogen in the Rosaceae family, numerous studies have focused on the functions and signaling pathways induced by harpins in plants [14,18,20,26,27,28,29,30]. The types of immunity primed by harpins include the common HR, through which harpin immunity was discovered, and non-HR immunity (Table 2). Typical HR-related cell death and non-HR defense responses, such as bursts in ROS production and increased expression of pathogenesis-related genes, callose deposition and phytohormone production, have been observed in harpin-treated plants [14,31,32,33,34].

### 2.2. Action Mechanisms Triggered by Harpins in Plants

Several possible explanations for harpin-induced HR are discussed herein. First, normal physiological cell membrane functions may be disrupted by harpins. Specifically, harpins bind to the plasma membrane of plant cells, which activates subsequent immune responses, and several harpins, such as HrpZ_Psph_ and PopA, form pores in the plasma membrane that enable ion conduction [25,35,36,37]. Second, mitochondrial function may be indirectly/directly disrupted by harpins, triggering mitochondrion-dependent programmed cell death in plants; for example, mitochondrial electron transport was inhibited by HrpN, which reduced ATP synthesis in tobacco. HrpZ1 induced the rapid release of cytochrome c from mitochondria into the cytosol, which led to the accumulation of ROS in *Arabidopsis thaliana.* RipX (previously named PopA) interacts with mitochondrial ATP synthase F1 subunit alpha (ATPA) and represses the transcription of *atpA* in *Nicotiana benthamiana* [29,38,39]. Third, harpins induce the expression of HR-related genes and activate MAPK signaling cascades [19,33,40,41,42].

Several membrane-localized harpin targets in plants have been identified, and their discovery has clarified harpin mechanisms of action. For example, a small transmembrane protein in apple (*Malus x domestica*) HrpN-interacting protein from *Malus* (HIPM) and its homologue in *Arabidopsis* (AtHIPM) have been found to interact with the harpin HrpN, and this interaction has been shown to be critical to susceptibility to *E. amylovora* [28,43]. The N-terminal 198 amino acids of HrpN were required for the HrpN protein interaction with HIPM. Further physiological analyses showed that the *atHIPM* knockout mutant was slightly larger than the wild type. All these results indicated that AtHIPM negatively regulates HrpN functions [28]. HIPM also interacts with oxygen-evolving enhancer-like protein (MdOEE), which may affect photosynthesis and subsequent ROS production [43]. The HIPM induction of this signaling axis may explain the mechanism through which harpins affect plant growth and immunity. In addition to HIPM, plasma membrane intrinsic protein (PIP) is a plasma membrane harpin sensor. The aquaporin AtPIP1;4, which is involved in the translocation of H_2_O_2_ from apoplasts to the cytoplasm to induce subsequent immune responses and membrane permeability of CO_2_ and H_2_O, functions as a sensor of Hpa1 in *A. thaliana*. Upon Hpa1 treatment, the interaction between Hpa1 and AtPIP1;4 led to increased photosynthesis to promote plant growth, suggesting that harpins induce the AtPIP1;4 signaling pathway to promote plant growth and trigger immune responses [26,44].

### 2.3. Plant Response to Harpins Promotes Biotic and Abiotic Resistance and Growth

Treatment with harpins contributes to pathogen resistance in plants (Table 2), which is an attribute with practical usefulness in agriculture. For example, the application of Messenger (a commercial product that contains Harpin (Ea) as the main active component), harpin (*Axiom*, Rx Green Technologies) and HpaXpm reduced the severity of diseases caused by *Phytophthora infestans* and *Botrytis cinerea, Pythium aphanidermatum,* and *Tobacco mosaic virus* (TMV) in tomato, hemp seedlings, and tobacco, respectively [14,19,45,46]. Transgenic plants expressing harpins, e.g., transgenic soybean expressing *hrpZpsta* from *P. syringae* pv. *tabaci* and *hrf2* from *Xanthomonas oryzae* pv. *oryzicola,* tobacco expressing *ripX* and *hpa1*, and transgenic cotton expressing *hpa1_Xoo_* from *X. oryzae* pv. *oryzae* have shown increased immune responses [29,30,31,47,48].

In addition to increases in immune responses and pathogen resistance, harpins induce other physiological activities, such as growth and development, tolerance to drought and high salinity (Table 2) [31,49,50,51,52,53,54]. Specifically, treatment with HpaXpm promoted increased root length and fresh weight in *A. thaliana*, and the expression of *SSB_Xoc_* and *Hpa1* in *N. benthamiana* promoted increased root length and plant growth [19,31,49]. This harpin-promoted growth may have been related to an increase in photosynthesis due to increased chlorophyll levels, CO_2_ conduction rates, and greater changes in the levels of phytohormones, e.g., ethylene (ET), gibberellin (GA), and salicylic acid (SA) [14,18,26,27,31,53]. Notably, exogenous PopW (a harpin from *Ralstonia solanacearum* ZJ3721) treatment led to greater drought resistance in *Medicago sativa* L. and tomato, and this effect was related to multiple mechanisms, such as the action of phytohormones, e.g., abscisic acid (ABA), GA, SA, and drought-related gene expression [15,52].

A particular partial harpin sequence has been shown to be sufficient for harpin function, although fragments are different in different harpins. Specifically, three heptads in the N-terminal coiled-coil domain of Hpa1 from *X. oryzae* pv. *Oryzae*, a 23-amino acid fragment of HpaG from *Xanthomonas axonopodis* pv. *glycines*, a 24-amino acid peptide in the HrpZ protein of *P. syringae,* were sufficient to initiate HR in plants [31,55,56]. Further studies showed that this particular segment of harpins induced a more robust response than that induced by harpins [22]. All these results suggest that certain harpin sequences are essential for the plant response, which may indicate that these elicitors can be leveraged to induce plant resistance to pathogens.

Different harpins show antagonistic effects. For example, HrpW(ea) from *E. amylovora* administered at subnanomolar concentrations decreased the defense responses that had been triggered by another harpin from this bacterium, HrpN(ea). This result may be attributable to the opposing anion channel changes triggered by HrpW(ea) and HrpN(ea) [57].

Studies have mainly focused on the architectures of harpins and the functions and action mechanisms involved in microbes and host plants for 30 years. Practical application of harpins has been successful; i.e., Messenger was registered in the United States and allowed to use on all crops in 2000; now, it has been used in many countries. More effective harpins remain to be used efficiently in practice and play more roles in agriculture and gardening.

**Table 2:** Involvement of proteinaceous elicitors in immune responses and abiotic resistance in plants.

Groups	Elicitors/Microbes	Plants	Treatments	Phytohormones	HR	Increase Resistance to Pathogens	Growth	Abiotic Resistance	References
Harpin	Harpin/*Erwinia amylovora*	*N. benthamiana*	infiltration	-	yes	-	-	-	[20]
	Harpin_Psph_/*Pseudomonas syringae* pv. *phaseolicola*	*N. benthamiana*	infiltration	-	yes	-	-	-	[35]
	PopW/*Ralstonia* *solanacearum*	*Medicago sativa* L.	-	ABA, GA, JA, SA, IAA	-	-	-	drought	[52]
	Hpa1/*Xanthomonas oryzae* pv. *oryzae*	*N. benthamiana*	*agrobacterium*-mediated transformation	-	yes (infiltration)	yes	yes	drought	[31]
	PopW/*Ralstonia solanacearum*	*Solanum lycopersicum* L.	foliar application	ABA	-	-	-	drought	[15]
	RipX(PopA)/*Ralstonia solanacearum*	*N. benthamiana*	infiltration	-	yes	-	-	-	[29]
	HpaXpm/*Xanthomonas* *phaseoli* pv. *manihotis*	*N. benthamiana*	infiltration	-	yes	yes (spraying)	-	-	[19]
	HpaXpm/*Xanthomonas* *phaseoli* pv. *manihotis*	*A. thaliana*	soak	-	-	-	yes	-	[19]
	SSB_Xoc_/*X. oryzae* pv. *oryzicola*	*N. benthamiana*	*agrobacterium*-mediated transformation	-	-	yes	yes	salt	[49]
	HrpZpsta/*P.* *syringae* pv. *tabaci*	*Glycine max*	*agrobacterium*-mediated transformation	-	-	yes	-	-	[47]
NLPs	CgNLP1/*Colletotrichum gloeosporioides*	*A. thaliana*	*agrobacterium*-mediated transformation	--	-	yes	-	-	[58]
	BsNep1/*Botrytis squamosa*	*N. benthamiana*	infiltration	--	yes	-	-	-	[59]
	BcNep1/*Botrytis cinerea*	*Allium cepa*	infiltration	--	yes	-	-	-	[59]
	VmNLP2/*Valsa mali*	*N. benthamiana*	agroinfiltration	--	yes	-	-	-	[11]
	VmNLP2/*Valsa mali*	apple	infiltration	--	yes	-	-	-	[11]
	PiNPP1.1/ *Phytophthora infestans*	*N. benthamiana*	agroinfiltration and infiltration	--	yes	-	-	-	[60]
	DserNEP1 and DserNEP2/*Diplodia seriata*	*Vitis vinifera*	dip and infiltration	--	yes	-	-	-	[61]
	PeNLP1 and PeNLP2/ *Penicillium expansum*	*N. benthamiana*	agroinfiltration	--	yes	-	-	-	[62]
	CoNLP1/*Colletotrichum orbiculare*	Several *Cucurbitaceae* cultivars	infiltration **	--	-	yes	-	-	[63]
	MoNLP1, MoNLP2 and MoNLP4/*Magnaporthe oryzae*	*N. benthamiana*	agroinfiltration	--	yes	-	-	-	[64]
	NLP_Pya_/*Pythium* *aphanidermatum*	*N. benthamiana*	infiltration	--	yes	-	-	-	[65]
	NLP_Pya_/*Pythium* *aphanidermatum*	*A. thaliana*	infiltration	--	yes	-	-	-	[65]
	NLP_Pya_/*Pythium* *aphanidermatum*	*Phalaenopsis amabilis*	infiltration	--	yes	-	-	-	[65]
	NLP_Pp_/*Phytophthora parasitica*	*A. thaliana*	infiltration	--	yes	-	-	-	[65]
Elicitin	INF1/*Phytophthora infestans*	*N. benthamiana*	infiltration	-	yes	-	-	-	[66]
	β-CRY/*Phytophthora cryptogea*	Three *Solanum* spp. genotypes	soak	ET, JA and JA–Ile	-	yes	-	-	[67]
	INF1/*Phytophthora infestans*	*N. benthamiana*	agroinfiltration	-	yes	-	-	-	[68]
	INF1/*Phytophthora infestans*	*N. benthamiana*	agroinfiltration	-	yes	-	-	-	[69]
	INF1/*Phytophthora infestans*	*Solanum microdontum*	agroinfection	-	yes	-	-	-	[70]
	Quercinin/*Phytophthora quercina*	*N. benthamiana*	cells soak	ET	yes	-	-	-	[71]
	Cryptogein/*Phytophthora cryptogea*	*N. benthamiana*	place onto the fresh wound	-	yes	yes	-	-	[72]
	Capsicein/*Phytophthora capsici*	*N. benthamiana*	place onto the fresh wound	-	yes	yes	-	-	[72]
	PoEli8/*Pythium oligandrum*	*N. benthamiana*, tomato, and pepper	infiltration	-	yes	yes	-	-	[73]

Note: Infiltration refers to infiltration by recombinant protein or cell extraction of recombinant *E. coli* and/or host pathogens. Phytohormones in the table refers to changes in phytohormone production. HR in the table refers to phenotype of plant. ** refers to infiltration of *Colletotrichum orbiculare* with NLP1 constitutive expression. - refers to not mentioned. -- refers to the changes in ethylene are not discussed in this table. Abbreviations: JA: jasmonic acid; IAA: indole-3-acetic acid; JA-Ile: jasmonic acid–isoleucine.

## 3. NLPs

Necrosis- and ethylene-inducing peptide 1 (Nep1)-like proteins (NLPs) constitute another group of proteinaceous elicitors widely secreted by bacteria, fungi, and oomycetes. NLPs contain characteristic NPP1 domains (PF05630) and are involved in multiple processes such as virulence, conidiospore production, formation of appressoria, abiotic stresses in microbes and triggering immune responses in plants [11,13,58,74,75]. On the basis of their impacts on host plants, NLPs are classified into two forms: cytotoxic to eudicot and monocot plants and noncytotoxic [59,65,76]. Cytotoxic NLPs are expressed when a pathogen converts from biotrophy into necrotrophy and function as pore-forming toxins (PFTs), and these NLPs bind to the terminal monomeric hexose moieties of glycosyl inositol phosphoryl ceramide (GIPC) to cause cytolysis [65,76,77,78]. Noncytotoxic NLPs induce an immune response without cytolysis. Since the first NLP protein, Nep1, was characterized in culture filtrates of *Fusarium oxysporum* f. sp. *erythroxyli* in 1995, many advances have been made to reveal the function and action mechanism of NLPs [79].

### 3.1. Taxonomy of NLPs

NLPs have been classified into types I, II, and III, on the basis of their amino acid sequences. Type I NLPs are the most abundant NLPs and can be found in bacteria, fungi, and especially oomycetes. Type II NLPs are found in bacteria, fungi and several oomycetes. Type I and type II NLPs have been characterized based on the number of cysteine residues they carry because cysteine is essential for their activity [76,80]. Type I NLPs usually carry 2 cysteine residues, which form one disulfide bridge, while type II NLPs generally harbor a second disulfide bridge, which is not required for necrosis induction [76]. Type II NLPs harbor a calcium-binding motif, which is essential for their cytotoxicity [81]. Type III NLPs, which exist exclusively in ascomycete fungi, are less conserved, with most NLPs harboring six cysteine residues [76,81]. Different species encode considerably different numbers of NLPs (including both cytotoxic and noncytotoxic types), ranging from only one or two in most bacteria to dozens in oomycetes, suggesting horizontal gene transfer in the evolution of NLPs [76].

Most NLPs are small proteins (which are approximately 25 kDa). They are single-domain (NPP1 domain, PF05630) proteins with signal peptides (SPs) on the N-terminus, and the NPP1 domain carries a conserved -HRH-W- fragment, which is important in the coordination of divalent cations and contributes to typical NLP features and physiological functions [76,82,83]. The protein is localized to the extracellular space through the classical secretion route and is further processed [76].

### 3.2. Involvement of NLPs in Immune Responses in Plants

As mentioned above, NLPs are involved in multiple processes in both microbes and plants. In this subsection, we focus on their effects on host plants (Table 2). Treatments with cytotoxic NLPs induce rapid cytolysis and result in plant cell necrosis [65,82]. In addition to NLPs functioning as PFTs, noncytotoxic NLPs and conserved fragments function as PAMPs/MAMPs to prime innate immunity in plants. Exogenous NLP or NLP fragment treatment or ectopic expression of NLP or conserved fragments (nlp20/nlp24) triggers plant immune responses, such as the expression of immune-responsive genes, activation of MAPK signaling cascades, bursts in ROS production, induction of SA, and enhanced plant immunity against pathogens in addition to necrosis and ethylene-induction [13,63,65,74,83,84,85].

### 3.3. Action Mechanisms of NLPs in Plant Immune Responses

The cytolytic mechanism triggered by cytotoxic NLPs has been described. As mentioned above, cytotoxic NLPs target and bind to the terminal hexose residues of glycosylinositol phosphorylceramide (GIPC) sphingolipids, which vary in total amount and concentration among different plants, which are located at the outer leaflet of the envelope membrane in plants [59,65]. The binding of GIPC-NLP is driven by electrostatic interactions, which can be strengthened in the presence of sterols and results in NLP conformational changes [65,77,86]. After binding, NLPs form functional oligomers and then form shallow transient pores that leak small molecules [77,87]. Tryptophan155 at the bottom of loop L3, an important amino acid involved in the NLP-GIPC interaction in model NLP_Pya_, was identified by mutation and crystallization [65,82,86,88]. Moreover, differences between cytotoxic and noncytotoxic NLPs have been identified by mutation and crystallization analysis and have been attributed to hydrophobic amino acid residues in the Lc1, L2 and L3 loops of NLPs [86].

**Figure 1:**
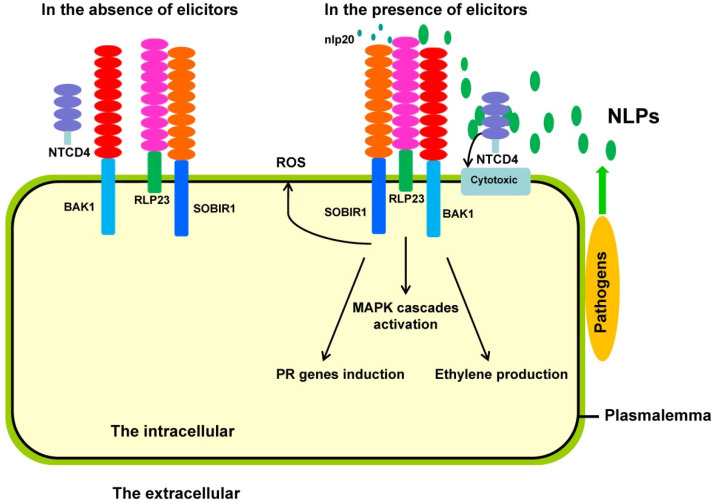
The BAK1–RLP23–SOBIR1 complex recognizes NLP peptides and mediates immune responses. RLP23 and SOBIR1 complex together and are isolated from BAK1 without NLP peptide perception. NLP peptides from pathogens are perceived by RLP23 and form tripartite receptor complexes of BAK1–RLP23–SOBIR1, which initiate downstream immune responses, including ROS burst, MAPK cascade activation, PR gene transcription and ethylene production. NTCD4, an LRR-only protein, promotes NLP oligomerization that induces pore formation on the plasma membrane, which is cytotoxic and triggers cell death.

In addition to sphingolipids as NLP receptors, protein receptors of NLPs in plants have been identified. Specifically, in *A. thaliana*, a leucine-rich repeat (LRR) receptor named RLP23 has been reported. RLP23 associates with the LRR-receptor kinase (LRR-RK) SOBIR1 in the absence of NLPs. Upon ligand binding, another LRR-RK, BAK1, binds to form a tripartite complex and induces intracellular innate immune responses. In this complex, the LRR domain is essential to the function of RLP23 (Figure 1) [89]. Unlike its positive roles in immune responses triggered by flg22, the convergent and central immune hub BIK1 plays a negative role in the immune responses induced by NLPs in *A. thaliana* [90]. The immune responses induced by NLPs were milder and slower than those induced by flg22 [90]. Furthermore, an LRR-only protein, NTCD4, has been reported to be involved in NLP oligomerization, which is an important step in pore formation caused by NLPs in *A. thaliana* [91]. Although involvements, action mechanisms and differences between cytotoxic and noncytotoxic NLPs in plant immunity have been elucidated, there is a lack of practical applications in agriculture and gardening.

## 4. Elicitins

Elicitins are small elicitors secreted by oomycetes (particularly *Phytophthora* and *Pythium*) that trigger necrosis and immune responses in a variety of plants, especially in tobacco and certain *Brassicaceae* species, which are the main sources of vegetables [92,93]. Since the first elicitins, cryptogein and capsicein (both are approximately 10 kDa), were identified in *Phytophthora cryptogea* and *Phytophthora capsici*, respectively, dozens of elicitins, e.g., parasiticein and INF1, have been identified [72,92]. Similar to harpins, elicitin structures are highly conserved with no similarity to plant proteins, which makes them good elicitors [94].

### 4.1. Taxonomy of Elicitins

According to a phylogenetic analysis, elicitins were divided into four elicitin (ELI) and 13 elicitin-like (ELL) clades, in which ELI proteins shared a highly conserved 98-amino-acid domain (SMART 01187, PFAM 00964) with at least 66% sequence identity and contain six cysteine residues that form three disulfide bridges, while ELL proteins showed more diversity in terms of the length of the elicitin domain and cysteine spacing patterns; although similar to ELI proteins, they carry six cysteine residues [93,95]. ELI and ELL proteins carry of a signal peptide at the N-terminus, an elicitin domain and variable C-terminal domains, which tend to be enriched with threonine, serine, and proline residues, except for the ELI-1 clade proteins [93,94]. Even though elicitins share high sequence similarity, they differ in net charge, which is the basis for classifying them into acidic α-elicitins and basic β-elicitins; among these proteins, β-elicitins show 100-fold more necrosis-inducing effects than α-elicitins. The difference results from the difference in amino acid residues at position 13, with α-elicitins harboring a valine residue and β-elicitins harboring a lysine residue. The residue at position 13 not only determines the isoelectric point (pI) and induces necrosis but also participates in ligand/receptor binding [96,97,98,99]. The numbers of ELI and ELL genes vary among species, with each gene showing differential expression patterns and HR-inducing activities [100,101].

### 4.2. Involvement and Action Mechanisms of Elicitins in Plant Immune Responses

Elicitins triggered HR and immune responses, e.g., the burst of ROS, electrolyte leakage, activation of MAPK cascades, induction of PR genes and phytohormone in tobacco, tomato, potato, pigeon pea, citrus, grapevine, pepper, oak and some *Brassicaceae* cultivars but not in many other herbaceous and woody plants (Table 2) [73,92]. The responses to elicitins in different plants were highly variable.

Upon LRR-receptor-like protein (RLP) elicitin response (ELR) recognition of the conserved elicitin domain, where leucine41 in the ω-loop region plays an important role, the constitutive ELR–SOBIR1 complex associates with the immune coreceptor BAK1/SERK3 in an elicitin-inducible manner; this interaction triggers immune responses, e.g., membrane depolarization, ion flux, an early-phase ROS burst, activation of MAPK signaling cascades, induction of pathogenesis-related genes, and an increase in phytoalexin and phytohormone levels and the necrosis rate [66,67,69,70,99,102,103,104]. As the first PRR to be identified, ELR recognizes a broad range of elicitins via their conserved elicitin domain [70]. In addition to ELR, an LRR-RLP REli from *N. benthamiana* has been identified recently as the receptor of PoEli8 from *Pythium oligandrum* [73].

Distinctive biphasic ROS accumulation has been observed in plants in which the immune response has been triggered by elicitins [92,93,105]. Moreover, early-phase ROS production was induced quickly after elicitin treatment via the existing NADPH oxidases RBOHA and RBOHB, while second-phase or late-phase ROS production occurred hours later and was induced by the de novo expression of the *rbohb* gene, which was triggered by the activation of the MAPK-WRKY7/8/9/11 signaling cascade, causing HR [93,106,107,108,109]. The involvement of nitric oxide (NO), which is partially mediated by the S-nitrosation of RBOHD1 and phosphorylation of MAPK kinases, has been reported in elicitin-triggered immune activation in plants [96,106,110,111,112]. The subsequent activation of PR genes, induction of cell death and increased SA, JA and ET levels have been detected [67,96].

Similar to harpins and NLPs, treatment with elicitins contributes to pathogen resistance in plants (Table 2), which would be practically useful in agriculture and gardening. For instance, the infiltration of PoEli8 improves resistance against *P. capsici* in tomato and pepper, and soaking in β-CRY improves resistance against *P*. *neolycopersici* in tomato [67,73].

## 5. Other Elicitors

In addition to the above-mentioned classes of elicitors, many other proteinaceous elicitors have been studied, e.g., RNA replicases from *Cucumber mosaic viru* and coat protein from turnip crinkle virus, CBEL from *Phytophthora parasitica* var. *nicotianae*; PeaT1 and Hrip1 from *Alternaria tenuissima*; MoHrip1, MoHrip2 and MoGluB from *Magnaporthe oryzae*; and BcGS1 and PebC1 from *Botrytis cinerea* [113,114,115,116,117,118,119,120,121,122,123,124]. A 2a polymerase of *Cucumber mosaic virus* induced HR responses in cowpea, and the induction was independent of its replicase activity [122]. The coat protein of turnip crinkle virus elicited HR responses in *Arabidopsis* [123]. When administered at a 150 nM concentration, CBEL, a 34 kDa glycoprotein elicitor located in the *P. parasitica* cell wall, displayed cellulose-binding and elicitor-like defense activity in plants as well as lectin-like activity, eliciting necrosis and defense-related gene expression in tobacco [121]. Further work showed that two intact cellulose-binding domains were sufficient and indispensable for the induction of a defense mode in the dicot plants tobacco and *A. thaliana* [125]. Despite showing low sequence homology, MoHrip1 and MoHrip2 were necessary for the virulence of fungi and induced defense responses via the SA and GA pathways, increased drought tolerance via the ABA pathway and increased plant growth [12,115,117,126,127,128,129]. PebC1 has been shown to be involved in increased plant growth, tolerance to drought, and disease and insect pest resistance [119,130,131,132].

## 6. Prospects and Challenges

In this mini-review, we summarized the taxonomy, structures and features of proteinaceous elicitors, described their involvement in plant immunity and responses to abiotic stresses, and discussed their mechanisms of action. Advances in the study of elicitors from pathogens that trigger plant immunity are helpful not only to increase the knowledge of plant-microbe interactions but also for practical use in agriculture and gardening. Although efforts have been made to identify the involvement and mechanisms of proteinaceous elicitors in plant immunity, the mechanisms remain to be discovered. Moreover, a wider range of elicitors remains to be identified, especially elicitors that trigger intense and persistent immune and abiotic stress responses in plants. The interactions of different elicitors also remain largely uncharacterized, even though several have been assessed and reported on certain interactions. What is the molecular basis by which elicitors cause different responses in hosts and nonhosts? What are the evolutionary dynamics of elicitors? Do elicitors cause an immune response in a wider range of plants than has been discovered to date? How can proteinaceous elicitors be made more effective and durable for commercial applications? Ultimately, a better understanding of proteinaceous elicitors in the plant response to stresses will help to establish healthier and more environmentally friendly agricultural practices worldwide.

## Figures and Tables

**Table 1:** Several carbohydrates and proteinaceous elicitors.

	Carbohydrates	Proteins/Peptides
Endogenous elicitors	xylan, oligo-galacturonic acid, …	glucanase, glutathione, …
Exogenous elicitors	peptidoglycan, chitin, glucan, lipopolysaccharides, …	flagellin (flg22), elongation factor Tu (EF-Tu), harpins, elicitins, NLPs, virus coat proteins, virus RNA replicases, …

## Data Availability

All data generated or analysed during this study are included in this article.

## References

[1] Damalas C.A., Eleftherohorinos I.G. (2011). Pesticide exposure, safety issues, and risk assessment indicators. Int. J. Environ. Res. Public Health.

[2] Guo J., Cheng Y. (2022). Advances in Fungal Elicitor-Triggered Plant Immunity. Int. J. Mol. Sci..

[3] Zhao Y., Zhu X., Chen X., Zhou J.M. (2022). From plant immunity to crop disease resistance. J. Genet. Genomics.

[4] Ngou B.P.M., Ding P., Jones J.D.G. (2022). Thirty years of resistance: Zig-zag through the plant immune system. Plant Cell.

[5] Wang Y., Pruitt R.N., Nurnberger T., Wang Y. (2022). Evasion of plant immunity by microbial pathogens. Nat. Rev. Microbiol..

[6] Zhou J.M., Zhang Y. (2020). Plant Immunity: Danger Perception and Signaling. Cell.

[7] Jones J.D., Dangl J.L. (2006). The plant immune system. Nature.

[8] Yuan M., Jiang Z., Bi G., Nomura K., Liu M., Wang Y., Cai B., Zhou J.M., He S.Y., Xin X.F. (2021). Pattern-recognition receptors are required for NLR-mediated plant immunity. Nature.

[9] Ngou B.P.M., Ahn H.K., Ding P., Jones J.D.G. (2021). Mutual potentiation of plant immunity by cell-surface and intracellular receptors. Nature.

[10] Lu C., Jiang Y., Yue Y., Sui Y., Hao M., Kang X., Wang Q., Chen D., Liu B., Yin Z. (2022). Glutathione and neodiosmin feedback sustain plant immunity. J. Exp. Bot..

[11] Liu J., Nie J., Chang Y., Huang L. (2021). Nep1-like Proteins from *Valsa mali* Differentially Regulate Pathogen Virulence and Response to Abiotic Stresses. J. Fungi..

[12] Nie H., Zhang L., Zhuang H., Yang X., Qiu D., Zeng H. (2019). Secreted protein MoHrip2 is required for full virulence of *Magnaporthe oryzae* and modulation of rice immunity. Appl. Microbiol. Biotechnol..

[13] Lian J., Han H., Chen X., Chen Q., Zhao J., Li C. (2022). *Stemphylium lycopersici* Nep1-like Protein (NLP) Is a Key Virulence Factor in Tomato Gray Leaf Spot Disease. J. Fungi..

[14] Sands L.B., Cheek T., Reynolds J., Ma Y., Berkowitz G.A. (2022). Effects of Harpin and Flg22 on Growth Enhancement and Pathogen Defense in *Cannabis sativa* Seedlings. Plants.

[15] Zhou X., Chen Y., Zhao Y., Gao F., Liu H. (2020). The application of exogenous PopW increases the tolerance of *Solanum lycopersicum* L. to drought stress through multiple mechanisms. Physiol. Mol. Biol. Plants.

[16] Choi M.S., Kim W., Lee C., Oh C.S. (2013). Harpins, multifunctional proteins secreted by gram-negative plant-pathogenic bacteria. Mol. Plant Microbe Interact..

[17] Wang X., Zhang L., Ji H., Mo X., Li P., Wang J., Dong H. (2018). Hpa1 is a type III translocator in *Xanthomonas oryzae* pv. oryzae. BMC Microbiol..

[18] Li X., Han L., Zhao Y., You Z., Dong H., Zhang C. (2014). Hpa1 harpin needs nitroxyl terminus to promote vegetative growth and leaf photosynthesis in *Arabidopsis*. J. Biosci..

[19] Liu Y., Zhou X., Liu W., Huang J., Liu Q., Sun J., Cai X., Miao W. (2020). HpaXpm, a novel harpin of *Xanthomonas phaseoli* pv. *manihotis*, acts as an elicitor with high thermal stability, reduces disease, and promotes plant growth. BMC Microbiol..

[20] Wei Z.M., Laby R.J., Zumoff C.H., Bauer D.W., He S.Y., Collmer A., Beer S.V. (1992). Harpin, elicitor of the hypersensitive response produced by the plant pathogen *Erwinia amylovora*. Science.

[21] Tarafdar P.K., Vedantam L.V., Podile A.R., Swamy M.J. (2013). Thermally stable harpin, HrpZPss is sensitive to chemical denaturants: Probing tryptophan environment, chemical and thermal unfolding by fluorescence spectroscopy. Biochimie.

[22] Liu Y., Zhou X., Liu W., Xiong X., Lv C., Zhou X., Miao W. (2018). Functional regions of HpaXm as elicitors with specific heat tolerance induce the hypersensitive response or plant growth promotion in nonhost plants. PLoS ONE.

[23] Charkowski A.O., Alfano J.R., Preston G., Yuan J., He S.Y., Collmer A. (1998). The *Pseudomonas syringae* pv. *tomato* HrpW protein has domains similar to harpins and pectate lyases and can elicit the plant hypersensitive response and bind to pectate. J. Bacteriol..

[24] Kim J.F., Beer S.V. (1998). HrpW of *Erwinia amylovora*, a new harpin that contains a domain homologous to pectate lyases of a distinct class. J. Bacteriol..

[25] Li J.G., Liu H.X., Cao J., Chen L.F., Gu C., Allen C., Guo J.H. (2010). PopW of *Ralstonia solanacearum*, a new two-domain harpin targeting the plant cell wall. Mol. Plant Pathol..

[26] Li L., Wang H., Gago J., Cui H., Qian Z., Kodama N., Ji H., Tian S., Shen D., Chen Y. (2015). Harpin Hpa1 Interacts with Aquaporin PIP1;4 to Promote the Substrate Transport and Photosynthesis in *Arabidopsis*. Sci. Rep..

[27] Li X., Han B., Xu M., Han L., Zhao Y., Liu Z., Dong H., Zhang C. (2014). Plant growth enhancement and associated physiological responses are coregulated by ethylene and gibberellin in response to harpin protein Hpa1. Planta.

[28] Oh C.S., Beer S.V. (2007). AtHIPM, an ortholog of the apple HrpN-interacting protein, is a negative regulator of plant growth and mediates the growth-enhancing effect of HrpN in *Arabidopsis*. Plant Physiol..

[29] Sun T., Wu W., Wu H., Rou W., Zhou Y., Zhuo T., Fan X., Hu X., Zou H. (2020). *Ralstonia solanacearum* elicitor RipX Induces Defense Reaction by Suppressing the Mitochondrial *atpA* Gene in Host Plant. Int. J. Mol. Sci..

[30] Niu L., Yang J., Zhang J., He H., Xing G., Zhao Q., Guo D., Sui L., Zhong X., Yang X. (2019). Introduction of the harpinXooc-encoding gene *hrf2* in soybean enhances resistance against the oomycete pathogen *Phytophthora sojae*. Transgenic Res..

[31] Ji Z.L., Yu M.H., Ding Y.Y., Li J., Zhu F., He J.X., Yang L.N. (2020). Coiled-Coil N21 of Hpa1 in *Xanthomonas oryzae* pv. *oryzae* Promotes Plant Growth, Disease Resistance and Drought Tolerance in Non-Hosts via Eliciting HR and Regulation of Multiple Defense Response Genes. Int. J. Mol. Sci..

[32] Peng J.L., Bao Z.L., Ren H.Y., Wang J.S., Dong H.S. (2004). Expression of harpin(xoo) in transgenic tobacco induces pathogen defense in the absence of hypersensitive cell death. Phytopathology.

[33] Li Y.R., Ma W.X., Che Y.Z., Zou L.F., Zakria M., Zou H.S., Chen G.Y. (2013). A highly-conserved single-stranded DNA-binding protein in *Xanthomonas* functions as a harpin-like protein to trigger plant immunity. PLoS ONE.

[34] Wang D., Wang B., Wang J., Wang S., Wang W., Niu Y. (2020). Exogenous Application of Harpin Protein Hpa1 onto *Pinellia ternata* Induces Systemic Resistance Against Tobacco Mosaic Virus. Phytopathology.

[35] Lee J., Klessig D.F., Nurnberger T. (2001). A harpin binding site in tobacco plasma membranes mediates activation of the pathogenesis-related gene *HIN1* independent of extracellular calcium but dependent on mitogen-activated protein kinase activity. Plant Cell.

[36] Racape J., Belbahri L., Engelhardt S., Lacombe B., Lee J., Lochman J., Marais A., Nicole M., Nurnberger T., Parlange F. (2005). Ca^2+^-dependent lipid binding and membrane integration of PopA, a harpin-like elicitor of the hypersensitive response in tobacco. Mol. Microbiol..

[37] Lee J., Klusener B., Tsiamis G., Stevens C., Neyt C., Tampakaki A.P., Panopoulos N.J., Noller J., Weiler E.W., Cornelis G.R. (2001). HrpZ(Psph) from the plant pathogen *Pseudomonas syringae* pv. *phaseolicola* binds to lipid bilayers and forms an ion-conducting pore in vitro. Proc. Natl. Acad Sci. USA.

[38] Xie Z., Chen Z. (2000). Harpin-induced hypersensitive cell death is associated with altered mitochondrial functions in tobacco cells. Mol. Plant Microbe Interact..

[39] Krause M., Durner J. (2004). Harpin inactivates mitochondria in *Arabidopsis* suspension cells. Mol. Plant Microbe Interact..

[40] Samuel M.A., Hall H., Krzymowska M., Drzewiecka K., Hennig J., Ellis B.E. (2005). SIPK signaling controls multiple components of harpin-induced cell death in tobacco. Plant J..

[41] Desikan R., Clarke A., Atherfold P., Hancock J.T., Neill S.J. (1999). Harpin induces mitogen-activated protein kinase activity during defence responses in *Arabidopsis thaliana* suspension cultures. Planta.

[42] Desikan R., Hancock J.T., Ichimura K., Shinozaki K., Neill S.J. (2001). Harpin induces activation of the *Arabidopsis* mitogen-activated protein kinases AtMPK4 and AtMPK6. Plant Physiol..

[43] Campa M., Piazza S., Righetti L., Oh C.S., Conterno L., Borejsza-Wysocka E., Nagamangala K.C., Beer S.V., Aldwinckle H.S., Malnoy M. (2019). *HIPM* Is a Susceptibility Gene of *Malus* spp.: Reduced Expression Reduces Susceptibility to *Erwinia amylovora*. Mol. Plant Microbe Interact..

[44] Tian S., Wang X.B., Li P., Wang H., Ji H.T., Xie J.Y., Qiu Q.L., Shen D., Dong H.S. (2016). Plant Aquaporin AtPIP1;4 Links Apoplastic H_2_O_2_ Induction to Disease Immunity Pathways. Plant Physiol..

[45] Fontanilla J.M., Montes M., De Prado R. (2005). Induction of resistance to the pathogenic agent *Botrytis cinerea* in the cultivation of the tomato by means of the application of the protein “Harpin”(Messenger). Commun. Agric. Appl. Biol. Sci..

[46] Fontanilla M., Montes M., De Prado R. (2005). Effects of the foliar-applied protein “Harpin(Ea)” (messenger) on tomatoes infected with *Phytophthora infestans*. Commun. Agric. Appl. Biol. Sci..

[47] Du Q., Yang X., Zhang J., Zhong X., Kim K.S., Yang J., Xing G., Li X., Jiang Z., Li Q. (2018). Over-expression of the *Pseudomonas syringae* harpin-encoding gene *hrpZm* confers enhanced tolerance to Phytophthora root and stem rot in transgenic soybean. Transgenic Res..

[48] Miao W., Wang X., Song C., Wang Y., Ren Y., Wang J. (2010). Transcriptome analysis of Hpa1Xoo transformed cotton revealed constitutive expression of genes in multiple signalling pathways related to disease resistance. J. Exp. Bot..

[49] Cao Y., Yang M., Ma W., Sun Y., Chen G. (2018). Overexpression of SSBXoc, a Single-Stranded DNA-Binding Protein From *Xanthomonas oryzae* pv. *oryzicola*, Enhances Plant Growth and Disease and Salt Stress Tolerance in Transgenic *Nicotiana benthamiana*. Front. Plant Sci..

[50] Chen L., Qian J., Qu S., Long J., Yin Q., Zhang C., Wu X., Sun F., Wu T., Hayes M. (2008). Identification of specific fragments of HpaG Xooc, a harpin from *Xanthomonas oryzae* pv. *oryzicola*, that induce disease resistance and enhance growth in plants. Phytopathology.

[51] Chen L., Zhang S.J., Zhang S.S., Qu S., Ren X., Long J., Yin Q., Qian J., Sun F., Zhang C. (2008). A fragment of the *Xanthomonas oryzae* pv. *oryzicola* harpin HpaG Xooc reduces disease and increases yield of rice in extensive grower plantings. Phytopathology.

[52] Demirkol G. (2021). PopW enhances drought stress tolerance of alfalfa via activating antioxidative enzymes, endogenous hormones, drought related genes and inhibiting senescence genes. Plant Physiol. Biochem..

[53] Dong H.P., Peng J., Bao Z., Meng X., Bonasera J.M., Chen G., Beer S.V., Dong H. (2004). Downstream divergence of the ethylene signaling pathway for harpin-stimulated *Arabidopsis* growth and insect defense. Plant Physiol..

[54] Dong Y., Li P., Zhang C. (2016). Harpin Hpa1 promotes flower development in *Impatiens* and *Parochetus* plants. Bot. Stud..

[55] Kim J.G., Jeon E., Oh J., Moon J.S., Hwang I. (2004). Mutational analysis of *Xanthomonas* harpin HpaG identifies a key functional region that elicits the hypersensitive response in nonhost plants. J. Bacteriol..

[56] Haapalainen M., Engelhardt S., Kufner I., Li C.M., Nurnberger T., Lee J., Romantschuk M., Taira S. (2011). Functional mapping of harpin HrpZ of *Pseudomonas syringae* reveals the sites responsible for protein oligomerization, lipid interactions and plant defence induction. Mol. Plant Pathol..

[57] Reboutier D., Bouteau F. (2008). Harpins and ion channels modulations: Many ways to die. Plant Signal. Behav..

[58] Yang G., Yang J., Zhang Q., Wang W., Feng L., Zhao L., An B., Wang Q., He C., Luo H. (2022). The Effector Protein CgNLP1 of *Colletotrichum gloeosporioides* Affects Invasion and Disrupts Nuclear Localization of Necrosis-Induced Transcription Factor HbMYB8-Like to Suppress Plant Defense Signaling. Front. Microbiol..

[59] Steentjes M.B.F., Herrera Valderrama A.L., Fouillen L., Bahammou D., Leisen T., Albert I., Nurnberger T., Hahn M., Mongrand S., Scholten O.E. (2022). Cytotoxic activity of Nep1-like proteins on monocots. New Phytol..

[60] Schumacher S., Grosser K., Voegele R.T., Kassemeyer H.H., Fuchs R. (2020). Identification and Characterization of Nep1-Like Proteins From the Grapevine Downy Mildew Pathogen *Plasmopara viticola*. Front. Plant Sci..

[61] Cobos R., Calvo-Pena C., Alvarez-Perez J.M., Ibanez A., Diez-Galan A., Gonzalez-Garcia S., Garcia-Angulo P., Acebes J.L., Coque J.J.R. (2019). Necrotic and Cytolytic Activity on Grapevine Leaves Produced by Nep1-Like Proteins of *Diplodia seriata*. Front. Plant Sci..

[62] Levin E., Raphael G., Ma J., Ballester A.R., Feygenberg O., Norelli J., Aly R., Gonzalez-Candelas L., Wisniewski M., Droby S. (2019). Identification and Functional Analysis of NLP-Encoding Genes from the Postharvest Pathogen *Penicillium expansum*. Microorganisms.

[63] Azmi N.S.A., Singkaravanit-Ogawa S., Ikeda K., Kitakura S., Inoue Y., Narusaka Y., Shirasu K., Kaido M., Mise K., Takano Y. (2018). Inappropriate Expression of an NLP Effector in *Colletotrichum orbiculare* Impairs Infection on *Cucurbitaceae* cultivars via Plant Recognition of the C-Terminal Region. Mol. Plant Microbe Interact..

[64] Fang Y.L., Peng Y.L., Fan J. (2017). The Nep1-like protein family of *Magnaporthe oryzae* is dispensable for the infection of rice plants. Sci. Rep..

[65] Lenarcic T., Albert I., Bohm H., Hodnik V., Pirc K., Zavec A.B., Podobnik M., Pahovnik D., Zagar E., Pruitt R. (2017). Eudicot plant-specific sphingolipids determine host selectivity of microbial NLP cytolysins. Science.

[66] Imano S., Fushimi M., Camagna M., Tsuyama-Koike A., Mori H., Ashida A., Tanaka A., Sato I., Chiba S., Kawakita K. (2021). AP2/ERF Transcription Factor NbERF-IX-33 Is Involved in the Regulation of Phytoalexin Production for the Resistance of *Nicotiana benthamiana* to *Phytophthora infestans*. Front. Plant Sci..

[67] Stary T., Satkova P., Piterkova J., Mieslerova B., Luhova L., Mikulik J., Kasparovsky T., Petrivalsky M., Lochman J. (2019). The elicitin β-cryptogein’s activity in tomato is mediated by jasmonic acid and ethylene signalling pathways independently of elicitin-sterol interactions. Planta.

[68] Turnbull D., Wang H., Breen S., Malec M., Naqvi S., Yang L., Welsh L., Hemsley P., Zhendong T., Brunner F. (2019). AVR2 Targets BSL Family Members, Which Act as Susceptibility Factors to Suppress Host Immunity. Plant Physiol..

[69] Domazakis E., Wouters D., Visser R.G.F., Kamoun S., Joosten M., Vleeshouwers V. (2018). The ELR-SOBIR1 Complex Functions as a Two-Component Receptor-Like Kinase to Mount Defense Against *Phytophthora infestans*. Mol. Plant Microbe Interact..

[70] Du J., Verzaux E., Chaparro-Garcia A., Bijsterbosch G., Keizer L.C., Zhou J., Liebrand T.W., Xie C., Govers F., Robatzek S. (2015). Elicitin recognition confers enhanced resistance to *Phytophthora infestans* in potato. Nat. Plants.

[71] Koehl J., Djulic A., Kirner V., Nguyen T.T., Heiser I. (2007). Ethylene is required for elicitin-induced oxidative burst but not for cell death induction in tobacco cell suspension cultures. J. Plant Physiol..

[72] Ricci P., Bonnet P., Huet J.C., Sallantin M., Beauvais-Cante F., Bruneteau M., Billard V., Michel G., Pernollet J.C. (1989). Structure and activity of proteins from pathogenic fungi *Phytophthora* eliciting necrosis and acquired resistance in tobacco. Eur. J. Biochem..

[73] Yang K., Wang Y., Zhao H., Shen D., Dou D., Jing M. (2022). Novel EIicitin from Pythium oligandrum Confers Disease Resistance against *Phytophthora capsici* in *Solanaceae* Plants. J. Agric. Food Chem..

[74] Yang C., Li W., Huang X., Tang X., Qin L., Liu Y., Xia Y., Peng Z., Xia S. (2022). SsNEP2 Contributes to the Virulence of *Sclerotinia sclerotiorum*. Pathogens.

[75] Magwanga R.O., Kirungu J.N., Lu P., Cai X., Zhou Z., Xu Y., Hou Y., Agong S.G., Wang K., Liu F. (2019). Map-Based Functional Analysis of the *GhNLP* Genes Reveals Their Roles in Enhancing Tolerance to N-Deficiency in Cotton. Int. J. Mol. Sci..

[76] Seidl M.F., Van den Ackerveken G. (2019). Activity and Phylogenetics of the Broadly Occurring Family of Microbial Nep1-Like Proteins. Annu. Rev. Phytopathol..

[77] Pirc K., Clifton L.A., Yilmaz N., Saltalamacchia A., Mally M., Snoj T., Znidarsic N., Srnko M., Borisek J., Parkkila P. (2022). An oomycete NLP cytolysin forms transient small pores in lipid membranes. Sci. Adv..

[78] Irieda H., Maeda H., Akiyama K., Hagiwara A., Saitoh H., Uemura A., Terauchi R., Takano Y. (2014). *Colletotrichum orbiculare* Secretes Virulence Effectors to a Biotrophic Interface at the Primary Hyphal Neck via Exocytosis Coupled with SEC_22_-Mediated Traffic. Plant Cell.

[79] Bailey B.A. (1995). Purification of a protein from culture filtrates of *Fusarium oxysporum* that induces ethylene and necrosis in leaves of *Erythroxylum coca*. Phytopathology.

[80] Fellbrich G., Romanski A., Varet A., Blume B., Brunner F., Engelhardt S., Felix G., Kemmerling B., Krzymowska M., Nurnberger T. (2002). NPP1, a *Phytophthora*-associated trigger of plant defense in parsley and *Arabidopsis*. Plant J..

[81] Oome S., Van den Ackerveken G. (2014). Comparative and functional analysis of the widely occurring family of Nep1-like proteins. Mol. Plant Microbe Interact..

[82] Ottmann C., Luberacki B., Kufner I., Koch W., Brunner F., Weyand M., Mattinen L., Pirhonen M., Anderluh G., Seitz H.U. (2009). A common toxin fold mediates microbial attack and plant defense. Proc. Natl. Acad Sci. USA.

[83] Yang K., Chen C., Wang Y., Li J., Dong X., Cheng Y., Zhang H., Zhai Y., Ai G., Song Q. (2022). Nep1-Like Proteins From the Biocontrol Agent *Pythium oligandrum* Enhance Plant Disease Resistance Independent of Cell Death and Reactive Oxygen Species. Front. Plant Sci..

[84] Dallal Bashi Z., Hegedus D.D., Buchwaldt L., Rimmer S.R., Borhan M.H. (2010). Expression and regulation of *Sclerotinia sclerotiorum* necrosis and ethylene-inducing peptides (NEPs). Mol. Plant Pathol..

[85] Tian H., Wu Z., Chen S., Ao K., Huang W., Yaghmaiean H., Sun T., Xu F., Zhang Y., Wang S. (2021). Activation of TIR signalling boosts pattern-triggered immunity. Nature.

[86] Lenarcic T., Pirc K., Hodnik V., Albert I., Borisek J., Magistrato A., Nurnberger T., Podobnik M., Anderluh G. (2019). Molecular basis for functional diversity among microbial Nep1-like proteins. PLoS Pathog..

[87] Pirc K., Albert I., Nurnberger T., Anderluh G. (2022). Disruption of plant plasma membrane by Nep1-like proteins in pathogen-plant interactions. New Phytol..

[88] Zaparoli G., Barsottini M.R., de Oliveira J.F., Dyszy F., Teixeira P.J., Barau J.G., Garcia O., Costa-Filho A.J., Ambrosio A.L., Pereira G.A. (2011). The crystal structure of necrosis- and ethylene-inducing protein 2 from the causal agent of cacao’s Witches’ Broom disease reveals key elements for its activity. Biochemistry.

[89] Albert I., Bohm H., Albert M., Feiler C.E., Imkampe J., Wallmeroth N., Brancato C., Raaymakers T.M., Oome S., Zhang H. (2015). An RLP23-SOBIR1-BAK1 complex mediates NLP-triggered immunity. Nat. Plants.

[90] Wan W.L., Zhang L., Pruitt R., Zaidem M., Brugman R., Ma X., Krol E., Perraki A., Kilian J., Grossmann G. (2019). Comparing *Arabidopsis* receptor kinase and receptor protein-mediated immune signaling reveals BIK1-dependent differences. New Phytol..

[91] Chen J.B., Bao S.W., Fang Y.L., Wei L.Y., Zhu W.S., Peng Y.L., Fan J. (2021). An LRR-only protein promotes NLP-triggered cell death and disease susceptibility by facilitating oligomerization of NLP in *Arabidopsis*. New Phytol..

[92] Derevnina L., Dagdas Y.F., De la Concepcion J.C., Bialas A., Kellner R., Petre B., Domazakis E., Du J., Wu C.H., Lin X. (2016). Nine things to know about elicitins. New Phytol..

[93] Janku M., Cincalova L., Luhova L., Lochman J., Petrivalsky M. (2020). Biological effects of oomycetes elicitins. Plant Prot. Sci..

[94] Jiang R.H., Tyler B.M., Whisson S.C., Hardham A.R., Govers F. (2006). Ancient origin of elicitin gene clusters in *Phytophthora* genomes. Mol. Biol. Evol..

[95] Boissy G., de La Fortelle E., Kahn R., Huet J.C., Bricogne G., Pernollet J.C., Brunie S. (1996). Crystal structure of a fungal elicitor secreted by *Phytophthora cryptogea*, a member of a novel class of plant necrotic proteins. Structure.

[96] Janku M., Jedelska T., Cincalova L., Sedlar A., Mikulik J., Luhova L., Lochman J., Petrivalsky M. (2022). Structure-activity relationships of oomycete elicitins uncover the role of reactive oxygen and nitrogen species in triggering plant defense responses. Plant Sci..

[97] O’Donohue M.J., Gousseau H., Huet J.C., Tepfer D., Pernollet J.C. (1995). Chemical synthesis, expression and mutagenesis of a gene encoding β-cryptogein, an elicitin produced by *Phytophthora cryptogea*. Plant Mol. Biol..

[98] Pleskova V., Kasparovsky T., Oboril M., Ptackova N., Chaloupkova R., Ladislav D., Damborsky J., Lochman J. (2011). Elicitin-membrane interaction is driven by a positive charge on the protein surface: Role of Lys13 residue in lipids loading and resistance induction. Plant Physiol. Biochem..

[99] Dokladal L., Oboril M., Stejskal K., Zdrahal Z., Ptackova N., Chaloupkova R., Damborsky J., Kasparovsky T., Jeandroz S., Zd’arska M. (2012). Physiological and proteomic approaches to evaluate the role of sterol binding in elicitin-induced resistance. J. Exp. Bot..

[100] Ponchet M., Panabieres F., Milat M.L., Mikes V., Montillet J.L., Suty L., Triantaphylides C., Tirilly Y., Blein J.P. (1999). Are elicitins cryptograms in plant-Oomycete communications?. Cell. Mol. Life Sci..

[101] Qutob D., Huitema E., Gijzen M., Kamoun S. (2003). Variation in structure and activity among elicitins from *Phytophthora sojae*. Mol. Plant Pathol..

[102] Lecourieux-Ouaked F., Pugin A., Lebrun-Garcia A. (2000). Phosphoproteins involved in the signal transduction of cryptogein, an elicitor of defense reactions in tobacco. Mol. Plant Microbe Interact..

[103] Wendehenne D., Lamotte O., Frachisse J.M., Barbier-Brygoo H., Pugin A. (2002). Nitrate efflux is an essential component of the cryptogein signaling pathway leading to defense responses and hypersensitive cell death in tobacco. Plant Cell.

[104] Liu Z.Q., Qiu A.L., Shi L.P., Cai J.S., Huang X.Y., Yang S., Wang B., Shen L., Huang M.K., Mou S.L. (2015). SRC2-1 is required in PcINF1-induced pepper immunity by acting as an interacting partner of PcINF1. J. Exp. Bot..

[105] Chaparro-Garcia A., Wilkinson R.C., Gimenez-Ibanez S., Findlay K., Coffey M.D., Zipfel C., Rathjen J.P., Kamoun S., Schornack S. (2011). The receptor-like kinase SERK3/BAK1 is required for basal resistance against the late blight pathogen *Phytophthora infestans* in *Nicotiana benthamiana*. PLoS ONE.

[106] Asai S., Ohta K., Yoshioka H. (2008). MAPK signaling regulates nitric oxide and NADPH oxidase-dependent oxidative bursts in *Nicotiana benthamiana*. Plant Cell.

[107] Ishihama N., Yamada R., Yoshioka M., Katou S., Yoshioka H. (2011). Phosphorylation of the *Nicotiana benthamiana* WRKY8 transcription factor by MAPK functions in the defense response. Plant Cell.

[108] Adachi H., Nakano T., Miyagawa N., Ishihama N., Yoshioka M., Katou Y., Yaeno T., Shirasu K., Yoshioka H. (2015). WRKY Transcription Factors Phosphorylated by MAPK Regulate a Plant Immune NADPH Oxidase in *Nicotiana benthamiana*. Plant Cell.

[109] Noirot E., Der C., Lherminier J., Robert F., Moricova P., Kieu K., Leborgne-Castel N., Simon-Plas F., Bouhidel K. (2014). Dynamic changes in the subcellular distribution of the tobacco ROS-producing enzyme RBOHD in response to the oomycete elicitor cryptogein. J. Exp. Bot..

[110] Saito S., Yamamoto-Katou A., Yoshioka H., Doke N., Kawakita K. (2006). Peroxynitrite generation and tyrosine nitration in defense responses in tobacco BY-2 cells. Plant Cell Physiol..

[111] Yamamoto-Katou A., Katou S., Yoshioka H., Doke N., Kawakita K. (2006). Nitrate reductase is responsible for elicitin-induced nitric oxide production in *Nicotiana benthamiana*. Plant Cell Physiol..

[112] Kulik A., Noirot E., Grandperret V., Bourque S., Fromentin J., Salloignon P., Truntzer C., Dobrowolska G., Simon-Plas F., Wendehenne D. (2015). Interplays between nitric oxide and reactive oxygen species in cryptogein signalling. Plant Cell Environ..

[113] Zhang W., Yang X., Qiu D., Guo L., Zeng H., Mao J., Gao Q. (2011). PeaT1-induced systemic acquired resistance in tobacco follows salicylic acid-dependent pathway. Mol. Biol. Rep..

[114] Kulye M., Liu H., Zhang Y., Zeng H., Yang X., Qiu D. (2012). Hrip1, a novel protein elicitor from necrotrophic fungus, *Alternaria tenuissima*, elicits cell death, expression of defence-related genes and systemic acquired resistance in tobacco. Plant Cell Environ..

[115] Chen M., Zeng H., Qiu D., Guo L., Yang X., Shi H., Zhou T., Zhao J. (2012). Purification and characterization of a novel hypersensitive response-inducing elicitor from *Magnaporthe oryzae* that triggers defense response in rice. PLoS ONE.

[116] Liu M., Liu X., Zeng H., Qiu D. (2013). Purification, crystallization and preliminary X-ray diffraction analysis of effector protein MoHrip2 from *Magnaporthe oryzae*. Acta Crystallogr. Sect. F Struct. Biol. Cryst. Commun..

[117] Chen M., Zhang C., Zi Q., Qiu D., Liu W., Zeng H. (2014). A novel elicitor identified from *Magnaporthe oryzae* triggers defense responses in tobacco and rice. Plant Cell Rep..

[118] Zhang Y., Zhang Y., Qiu D., Zeng H., Guo L., Yang X. (2015). BcGs1, a glycoprotein from *Botrytis cinerea*, elicits defence response and improves disease resistance in host plants. Biochem. Biophys. Res. Commun..

[119] Zhang Y., Yang X., Liu Q., Qiu D., Zhang Y., Zeng H., Yuan J., Mao J. (2010). Purification of novel protein elicitor from *Botrytis cinerea* that induces disease resistance and drought tolerance in plants. Microbiol. Res..

[120] Wang Y., Zhao Y., Wang X., Zhong L., Fan Q., Lan Z., Ye X., Huang Y., Li Z., Cui Z. (2021). Functional Characterization of the Novel Laminaripentaose-Producing beta-1,3-Glucanase MoGluB and Its Biocontrol of *Magnaporthe oryzae*. J. Agric. Food Chem..

[121] Mateos F.V., Rickauer M., Esquerre-Tugaye M.T. (1997). Cloning and characterization of a cDNA encoding an elicitor of *Phytophthora parasitica* var. *nicotianae* that shows cellulose-binding and lectin-like activities. Mol. Plant Microbe Interact..

[122] Hu Z., Zhang T., Yao M., Feng Z., Miriam K., Wu J., Zhou X., Tao X. (2012). The 2a protein of *Cucumber mosaic virus* induces a hypersensitive response in cowpea independently of its replicase activity. Virus Res..

[123] Kachroo P., Yoshioka K., Shah J., Dooner H.K., Klessig D.F. (2000). Resistance to turnip crinkle virus in Arabidopsis is regulated by two host genes and is salicylic acid dependent but NPR1, ethylene, and jasmonate independent. Plant Cell.

[124] Garcia J.A., Pallas V. (2015). Viral factors involved in plant pathogenesis. Curr. Opin. Virol..

[125] Gaulin E., Drame N., Lafitte C., Torto-Alalibo T., Martinez Y., Ameline-Torregrosa C., Khatib M., Mazarguil H., Villalba-Mateos F., Kamoun S. (2006). Cellulose binding domains of a *Phytophthora* cell wall protein are novel pathogen-associated molecular patterns. Plant Cell.

[126] Khan N.U., Liu M., Yang X., Qiu D. (2016). Fungal Elicitor MoHrip2 Induces Disease Resistance in Rice Leaves, Triggering Stress-Related Pathways. PLoS ONE.

[127] Wang Z., Han Q., Zi Q., Lv S., Qiu D., Zeng H. (2017). Enhanced disease resistance and drought tolerance in transgenic rice plants overexpressing protein elicitors from *Magnaporthe oryzae*. PLoS ONE.

[128] Lv S., Wang Z., Yang X., Guo L., Qiu D., Zeng H. (2016). Transcriptional Profiling of Rice Treated with MoHrip1 Reveal the Function of Protein Elicitor in Enhancement of Disease Resistance and Plant Growth. Front Plant Sci..

[129] Nie H.Z., Zhang L., Zhuang H.Q., Shi W.J., Yang X.F., Qiu D.W., Zeng H.M. (2019). The Secreted Protein MoHrip1 Is Necessary for the Virulence of *Magnaporthe oryzae*. Int. J. Mol. Sci..

[130] Basit A., Hanan A., Nazir T., Majeed M.Z., Qiu D. (2019). Molecular and Functional Characterization of Elicitor PeBC1 Extracted from *Botrytis cinerea* Involved in the Induction of Resistance against Green Peach Aphid (*Myzus persicae*) in Common Beans (*Phaseolus vulgaris* L.). Insects.

[131] Javed K., Humayun T., Humayun A., Wang Y., Javed H. (2021). PeaT1 and PeBC1 Microbial Protein Elicitors Enhanced Resistance against *Myzus persicae* Sulzer in Chili *Capsicum annum* L.. Microorganisms.

[132] Zhang Y., Yang X., Zeng H., Guo L., Yuan J., Qiu D. (2014). Fungal elicitor protein PebC1 from *Botrytis cinerea* improves disease resistance in *Arabidopsis thaliana*. Biotechnol. Lett..

